# EXPLORING HOW “TELE-SAVVY” INFLUENCES SELF-CARE BEHAVIORS FOR DEMENTIA FAMILY CAREGIVERS

**DOI:** 10.1093/geroni/igz038.3195

**Published:** 2019-11-08

**Authors:** Fayron Epps, Elizabeth Maloch, Patricia Griffiths, Ken Hepburn

**Affiliations:** 1 Emory University, Atlanta, Georgia, United States; 2 Emory University School of Medicine, Atlanta, Georgia, United States

## Abstract

Tele-Savvy is a 3-arm randomized controlled trial (RCT) of a psychoeducation intervention that equips dementia family caregivers with the knowledge and skills they need to provide care to their person, while also caring for themselves. This RCT is currently underway, with cohorts rotating through over a period of 12 months. The purpose of this presentation is to explore the effectiveness of Tele-Savvy (active) versus Healthy Living Intervention (attention control) or usual care (waitlist) on self-care behaviors among dementia family caregivers. We conducted semi-structured interviews with 16 caregivers after their initial participation in either the active, attention control, or usual care groups. Interviews elicited caregivers’ perceptions regarding the program’s influence on their self-care behaviors and engagement in self-care activities. The overall emerging theme for the family caregivers who participated in the Tele-Savvy and Healthy Living programs was “increased awareness of self-care activities”. Family caregivers in the Healthy Living program spoke mainly about engaging more in physical activities and improving their nutrition by eating healthier and keeping track of foods. Across all study groups, social engagement and having a support system were common self-care activities. Various limitations to engaging in self-care activities while in enrolled in the study included time and caregiving responsibilities. Suggestions were made for more respite services to become available to allow for family caregivers to engage in self-care activities. These findings provide researchers and practitioners with pertinent information to develop and refine programs for family caregivers to improve their self-care practices.

